# Supplementary material for: Patterns of Intron Gain and Loss in Fungi
Source: PLoS Biol. 2004 Nov 30;2(12):e422. doi: 10.1371/journal.pbio.0020422 (PMC532390; doi:10.1371/journal.pbio.0020422)
Supplement: Table S1 — Also available at http://genes.mit.edu/NielsenEtAl/. (4.3 MB ZIP). [file pbio.0020422.st001.zip › NielsenEtAl/html/27.html]

AN4559.1.NCU00075.1.MG10159.1.FG06723.1


```
 CLUSTAL W (1.82) Multiple Sequence Alignments - Introns Inserted


Sequence 1: MG10159.1	106 aa
Sequence 2: FG06723.1	105 aa
Sequence 3: NCU00075.1	105 aa
Sequence 4: AN4559.1	105 aa
Alignment Length: 106 aa
Number Identitical Residues: 71 aa
Alignment Score (without introns) 3081


MG10159.1 	MASTVGLVAGAAVAAFL0GRAGYVALRRSRGGVGAMGKAFYKG~GFENKMNQKEASLILS
NCU00075.1	MSSAVAIGAGVAVAAFL0GRAGLVAWRRSRGGVGALGKAFYKG~GFEPRMNKKEASLILS
FG06723.1 	MASVMAWGAGAAVAAFL0GRAGLVAWRRSRGGVGAMGKAFYKG~GFEAKMTKKEATLILS
AN4559.1  	MASALTLGLGVATAAFL0GRAGLVAYRRSKGGVNALGKAFYKG2GFEPRMNRREAALILE
          	*:*.:    *.*.**** **** ** ***:***.*:******* *** :*.::**:***.

MG10159.1 	LN2ERSITKDKVRKAHRTLMLLNHPDRGGSPYLATKINEAKELLEKQAS
NCU00075.1	LN2ERTITKDKIRKAHRTLMLLNHPDRGGSPYLATKVNEAKEFLEKSV-
FG06723.1 	LN2ERAITKDKVRKAHRTLMLLNHPDRGGSPYLATKVNEAKEFLDKNG-
AN4559.1  	LP2ERTLNKEKVRKKHRQLMLLNHPDRGGSPYLATKINEAKEFLDKHT-
          	*  **::.*:*:** ** ******************:*****:*:*
```
